# Transumbilical single-site laparoscopic treatment of primary splenic cyst in child: a rare case report and review of literature

**DOI:** 10.3389/fped.2024.1454487

**Published:** 2024-09-25

**Authors:** Meng Kong, Shuai Chen, Yuexia Bai, Yuxi Yan, Minggang Yi, Meiyun Wang, Hongzhen Liu, Jinhua Jia, Chuanyang Liu, Shisong Zhang

**Affiliations:** ^1^ Department of Pediatric Surgery, Children’s Hospital Affiliated to Shandong University, Jinan, China; ^2^ Department of Pediatric Surgery, Jinan Children’s Hospital, Jinan, China; ^3^ Department of Pathology, Children’s Hospital Affiliated to Shandong University, Jinan, China; ^4^ Department of Ultrasound, Children’s Hospital Affiliated to Shandong University, Jinan, China; ^5^ Department of Radiology, Children’s Hospital Affiliated to Shandong University, Jinan, China; ^6^ Child Health Department, Children’s Hospital Affiliated to Shandong University, Jinan, China

**Keywords:** splenic cyst, children, laparoscopy, single site, partial splenectomy

## Abstract

**Background:**

Splenic cysts are relatively rare benign tumors that are asymptomatic in most patients and are usually discovered incidentally by imaging. In our case, we report a splenic cyst in a child who underwent laparoscopic partial splenectomy.

**Case description:**

A 13-year-old boy was admitted to the hospital after an incidental finding of a splenic cyst on an abdominal ultrasound conducted 4 days prior. He was asymptomatic before admission. Upon admission, abdominal ultrasound and CT revealed a cystic lesion in the spleen, highly suspicious for a splenic cyst. Then, we used transumbilical single-site laparoscopic exploration and found a cyst measuring approximately 12 cm × 11 cm × 10 cm at the upper pole of the spleen, so we performed a partial splenectomy and diagnosed a primary epithelioid splenic cyst via postoperative pathology.

**Conclusions:**

Splenic cysts in children are very rare and can be treated conservatively in asymptomatic patients with a diameter of less than 5 cm, while surgery is required in symptomatic patients or those with a diameter greater than or equal to 5 cm. Transumbilical single-site laparoscopic partial splenectomy is a minimally invasive and effective treatment, especially for children.

## 1 Introduction

Splenic cysts are relatively rare clinically recognized splenic space-occupying lesions, with a prevalence of approximately 0.07% of all splenectomies and 0.5% of all splenectomies (1). Splenic cysts are categorized as parasitic or nonparasitic on the basis of the type of pathology, and approximately 60% of splenic cysts are parasitic in origin. Nonparasitic splenic cysts are further categorized as primary or secondary (2) (Table 1). Primary splenic cysts, also known as congenital splenic cysts, have a cyst wall lined with epithelial components and account for 10% of nonparasitic splenic cysts (4). It can occur at any age and most patients are clinically asymptomatic, usually by chance. However, it may cause abdominal discomfort, pain, or an abdominal mass (5), and in a very small number of patients, complications such as acute abdominal bleeding, infection, or rupture (6, 7) may also occasionally present as hypersplenism with elevated blood pressure (8). The diagnosis of primary splenic cysts relies on not only rely on clinical manifestations but also imaging, and histopathological examination is still the final means of diagnosis. At present, the treatment of primary splenic cysts is controversial. However, partial splenectomy may be the most effective method for preserving spleen function and preventing recurrence (9–11). Here, we report a case of a large primary splenic cyst in a child who had no symptoms and was found only by chance via abdominal color ultrasound. Our objective was to perform partial splenic resection assisted by transumbilical single-site laparoscopy, which not only preserves normal splenic tissue and function, but also has the advantages of less trauma, quick recovery and good cosmetic effects.

**Table 1:** Classification of non-parasitic cysts (3).

Primary	Secondary
Congenital	Traumatic
Mesothelial	Necrotic
Metaplastic	
Transitional	
Stratified squamous	
Neoplastic	
Angioma	
Haemangioma	
Lymphangioma	
Dermoid	

## 2 Case report

### 2.1 General information

A 13-year-old male was admitted to the hospital with an incidental finding of a cystic spleen on abdominal ultrasound, which had been present for 4 days. He had no fever, vomiting, abdominal distension, abdominal pain or any other discomfort before admission. There was no history of trauma, living in a parasite-endemic area, or family history of hereditary disease, and there was no history of animal contact.

### 2.2 Physical examination and auxiliary examination

The patient exhibited no jaundice, rash, or petechiae. Superficial lymph nodes were not palpable. The abdomen was flat, with no signs of gastrointestinal symptoms or abdominal wall varices. The abdomen was soft, without tenderness, rebound tenderness, or muscle rigidity. The liver was not palpable below the ribs, while the spleen extended 5 cm below the costal margin. A cystic mass approximately 10 cm × 10 cm, with poor mobility, was palpable in the left upper abdomen. Laboratory tests: Blood cell analysis revealed the following: white blood cell count, 5.73 × 10^9^/L; red blood cell count, 5.15 × 10^12^/L; hemoglobin volume, 134 g/L; and platelet count, 193 × 10^9^/L. Blood biochemistry revealed the following: total protein, 73.1 g/L; globulin, 28.2 g/L; the ratio of albumin to globulin, 1.6; total bilirubin, 12.1 μmol/L; and direct bilirubin, 5.0 μmol/L. Serum tumor marker levels were not abnormal.

**Figure 1:**
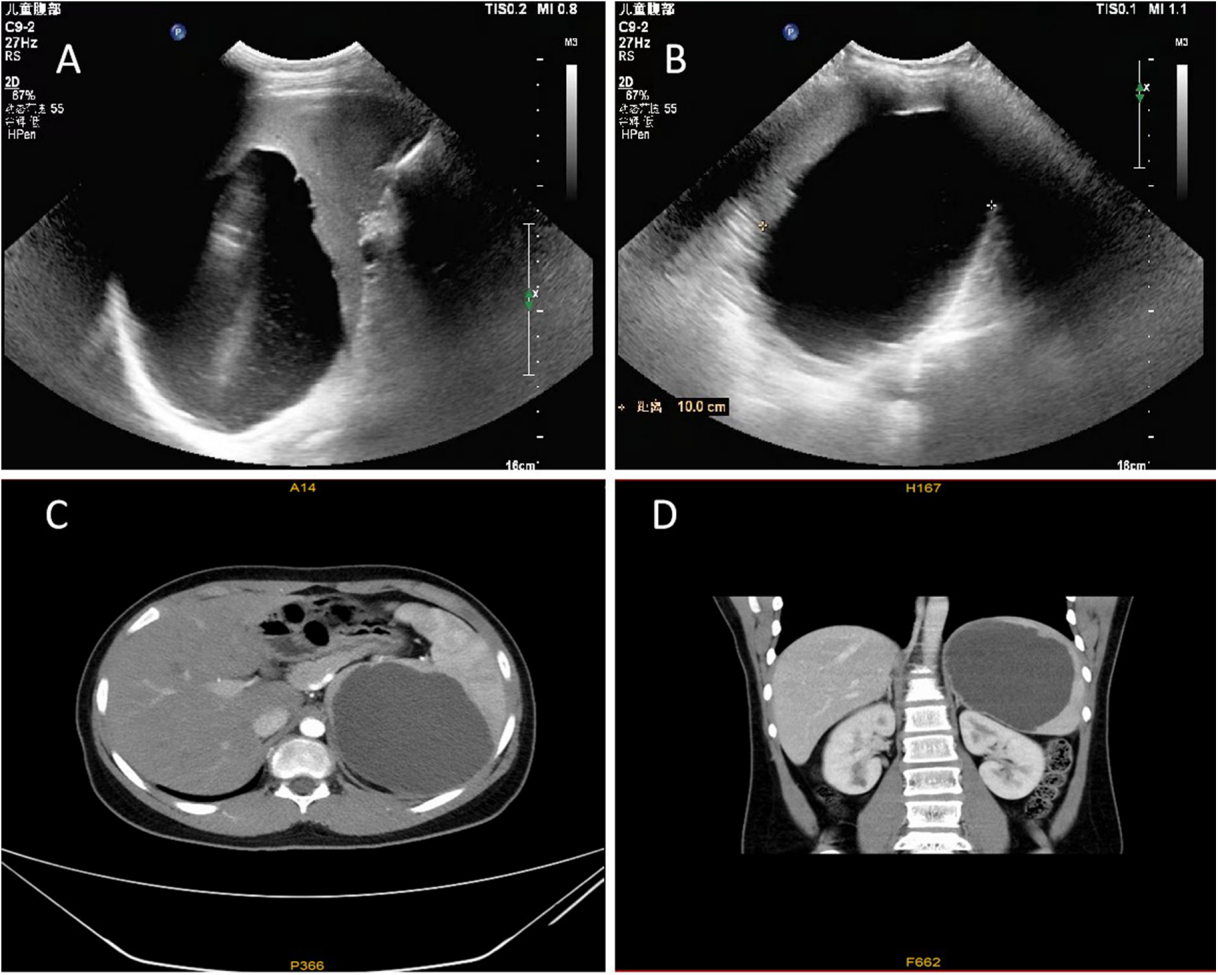
Ultrasound and CT imaging findings of splenic cyst. **(A)** Abdominal ultrasound findings showed a cystic echogenic area measuring approximately 11.4 cm × 11.0 cm × 10.0 cm explored at the upper pole of the spleen, with clear borders and poor internal translucency (Transverse section of the spleen). **(B)** Abdominal ultrasound showed the longitudinal section of the spleen. **(C)** Abdominal CT results showed an enlarged spleen with a cystic hypodense shadow measuring approximately 10.8 cm × 10.0 cm × 9.1 cm, with clear borders, and no obvious abnormal enhancement on enhancement scan. This figure shows a cross section of the spleen. **(D)** CT showed the coronal surface of the spleen.

Abdominal ultrasound revealed that the spleen was morphologically abnormal, measuring approximately 16.5 cm × 11.0 cm × 9.8 cm, and a cystic echogenic area measuring approximately 11.4 cm × 11.0 cm × 10.0 cm was detected in the parenchyma, with clear borders, poor internal translucency, and punctate echoes, suggesting a splenic cyst (Figures 1A,B). Abdominal CT (scanning + enhancement) revealed that the spleen was enlarged in size, and a cystic low-density shadow was observed within it, measuring approximately 10.8 cm × 10.0 cm × 9.1 cm, with clear borders, The enhancement scan did not show any obvious abnormal enhancement, which was consistent with the CT manifestation of the splenic cyst (Figures 1C,D). In addition, the morphology, size, and density of the other organs, such as the liver, biliary tract, pancreas, and the kidneys, were not abnormal, and the enhancement scan didn't reveal any obvious abnormal enhancement. The enhancement scan didn't show any abnormal.

### 2.3 Surgical procedure

Based on the patient's medical history, physical examination and auxiliary examination, we considered that the possibility of splenic cysts was high, and in view of the child's young age, only 13 years old, and the diameter of the cyst was greater than 5 cm and located in the upper pole of the spleen, therefore, we adopted the treatment of transumbilical single-site laparoscopic partial splenectomy.

**Figure 2:**
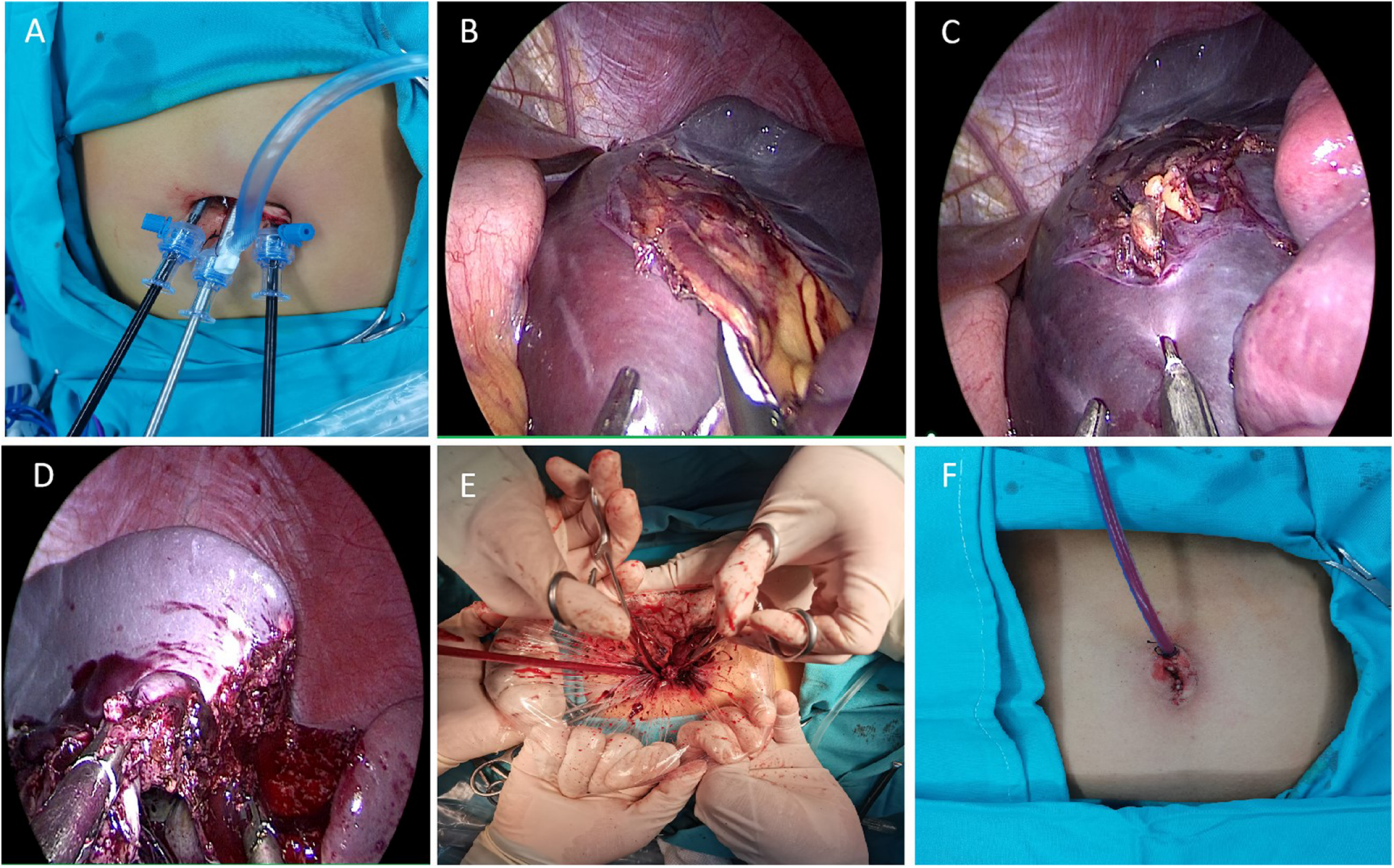
Method and procedure of transumbilical single site laparoscopic splenic cyst resection. **(A)** Trocar position in the umbilicus. **(B)** Exposure of the superior pole of the splenic vessels. **(C)** Puncture needle to aspirate fluid from the splenic cyst. **(D)** Ligasure resection of the upper pole of the spleen. **(E)** Removal of the splenic cyst from the umbilical incision. **(F)** Postoperative umbilical appearance.

The specific operation steps were as follows: (1) The child was placed in the supine position, with both lower limbs spread approximately 45°, in the head-high-foot-sole position, and the left lumbar pad was elevated approximately 15°. The preoperative urinary catheter and gastric tube were left in place, the laparoscopic monitor was placed on the outside of the patient's left shoulder, the operator was located between the patient's legs, and the person holding the mirror was located on the patient's right side. (2) A longitudinal umbilical incision of about 2.0 cm was made to keep the fascial layer intact and avoid air leakage during pneumoperitoneum. From the middle of the incision to both sides, the free subcutaneous tissue was expanded to the muscle layer. (3) A 5 mm trocar was placed in the center of the umbilicus to establish a CO_2_ pneumoperitoneum, with a pressure of 10 mmHg and a flow rate of 8 L/min, and the trocar was disposed of into the laparoscope as an observation hole. A 5 mm trocar was inserted into the left and right sides of the umbilical incision as the operation hole (Figure 2A). (4) The spleen was located in the left epigastric region and was visibly enlarged and purplish in color. The surgeon used Ligasure to open the left gastrocolic ligament. Then, the needle was inserted into the abdominal wall approximately 3 cm below the xiphoid process, and the greater curvature of the stomach near the fundus of the stomach to the abdominal wall was suspended to fully expose the splenic-gastric ligament and maintain moderate tension. (5) Ligasure was used to dissect the splenogastric ligament upwards to fully expose the spleen, and further exploration revealed a single enlarged cyst on the medial side of the upper pole of the spleen, measuring approximately 11.0 cm × 10.0 cm × 7 cm, with a localized dark blue color and unclear boundaries with the normal spleen. (6) The surface of the cyst, and the upper pole of the splenic artery was exposed. After the artery was completely removed from the upper pole of the spleen (Figure 2B), it was ligated with a silk thread and dissected, and the color of the upper pole of the spleen was clearly darker by ischemia. The spleen was then turned to the right, and the splenophrenic ligament and splenorenal ligament were severed by Ligasure. (7) Due to the large splenic cyst, the puncture needle was quickly inserted into the cyst through trocar on the left side of the umbilical cord during the operation (Figure 2C); approximately 500 ml of light yellow cyst fluid was aspirated, and a small amount of cyst fluid was taken for cytological examination. No parasitic infection was detected. (8) The upper pole of the spleen along the ischemic demarcation line between the upper and lower poles of the spleen was excised along with the cyst by Ligasure (Figure 2D), and the trauma was sufficiently hemostatic by cauterizing the wound with an electrocoagulation hook. (9) The excised spleen was placed into an extraction bag and cut into small pieces. After three trocars were removed, the tissue was cut lengthwise along the median umbilical incision to enlarge the umbilical incision, and the samples were extracted via oval forceps or hemostatic forceps (Figure 2E). (10) After reestablishing the pneumoperitoneum and rechecking that the splenic wound was free of active bleeding, one drain was left around the splenic wound, which was drained and fixed from the umbilical wound, and the incision was closed (Figure 2F). (11) Intraoperative bleeding was approximately 30 ml, no blood transfusion was performed, and the operation was successfully completed.

### 2.4 Patient postoperative conditions and pathological findings

**Figure 3:**
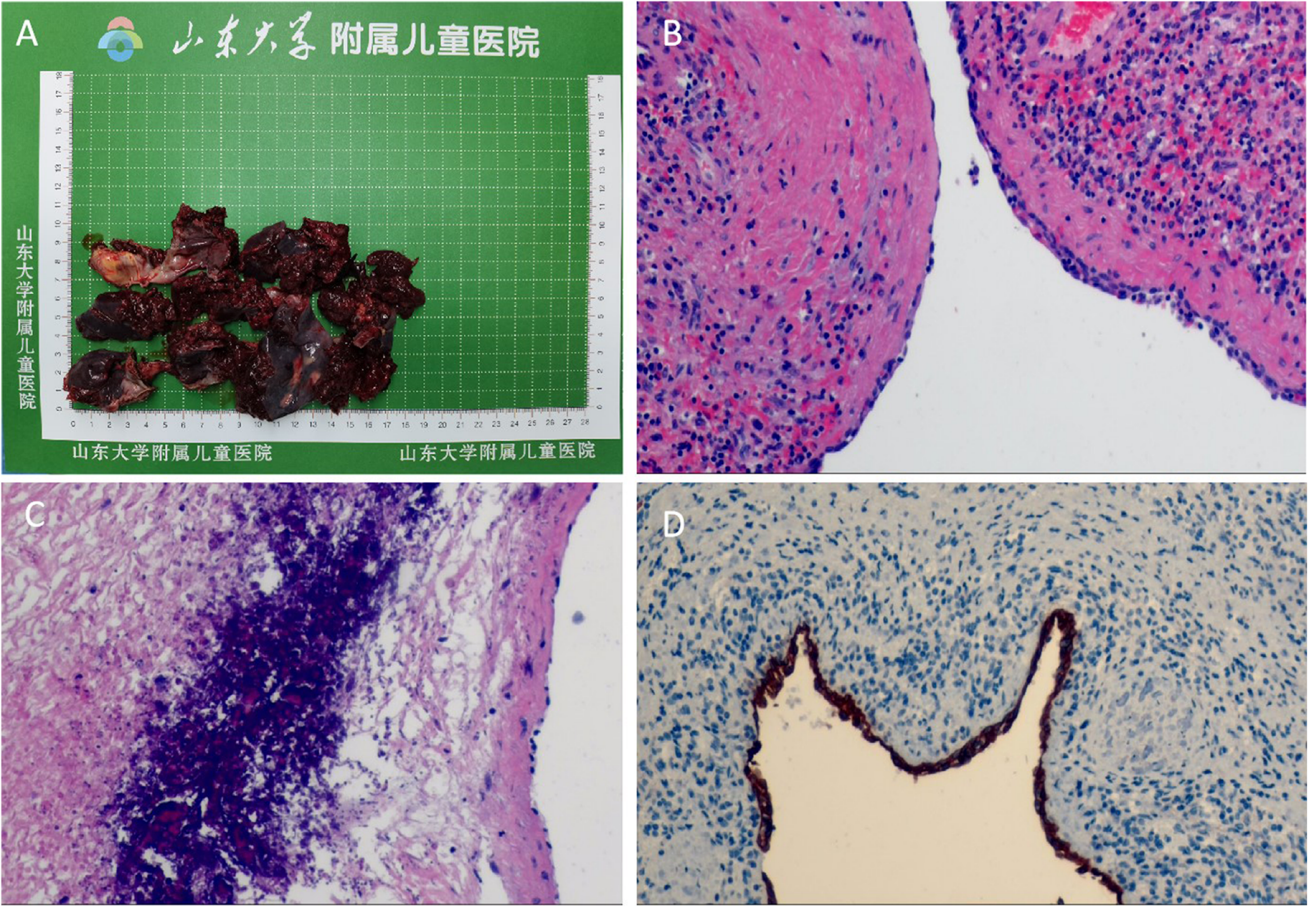
Gross appearance and histopathological findings of resected splenic cyst specimen. **(A)** The gross examination of the resected splenic cyst reveals a grayish-red surface that is smooth and uniform. Certain areas of the specimen exhibit a wall-like appearance typical of cystic structures. **(B)** HE staining showed primary epithelioid cyst lined with a single layer of cuboidal or flattened epithelium; ×100. **(C)** Fibrous tissue hyperplasia with focal calcification was seen in the splenic cyst; ×100. **(D)** Immunohistochemistry showed that the splenic cyst was lined with epithelial cells that were positive for CK; ×100.

**Figure 4:**
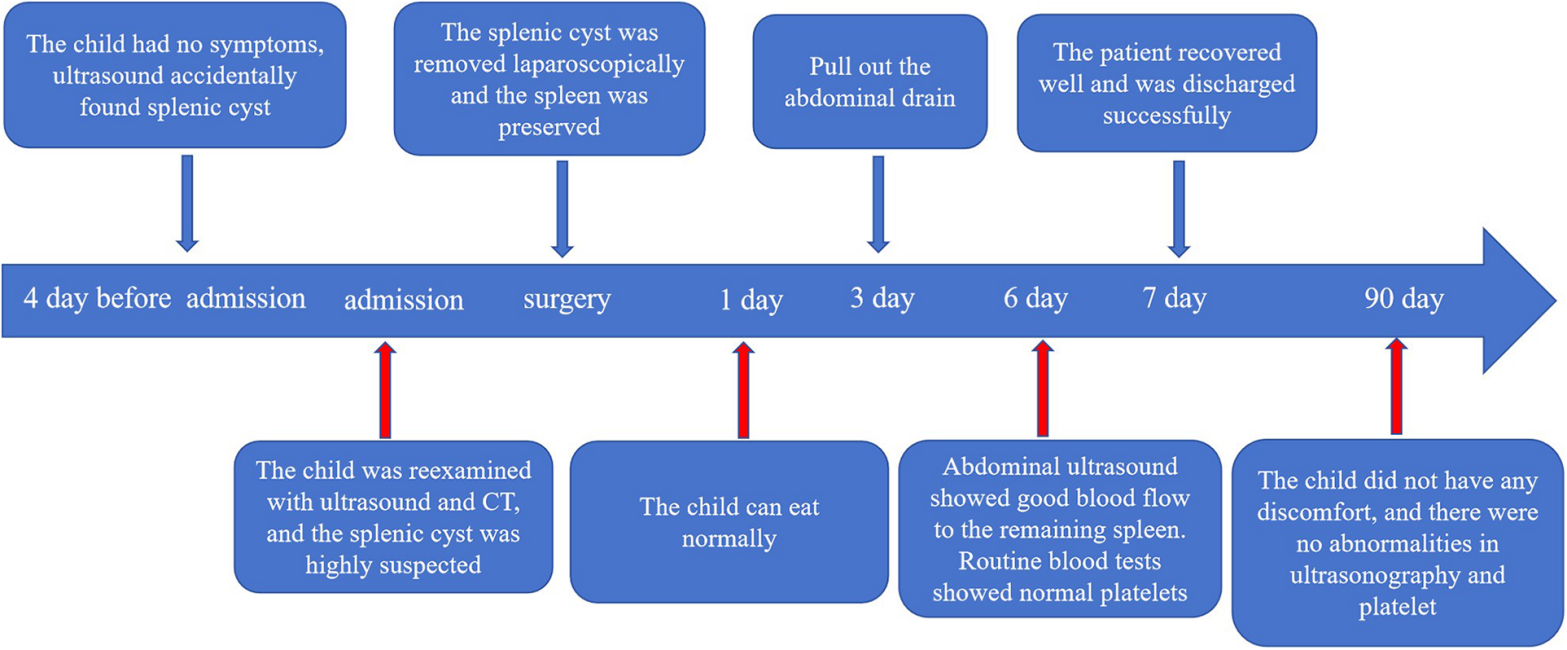
Complete timeline, including diagnosis, surgery, postoperative recovery, and follow-up.

The child started eating on the 1st postoperative day, the abdominal drain was removed on the 3rd day, and the blood cell analysis was repeated on the 6th day: red blood cell count 10.88 × 10^12^/L; and platelet count, 346 × 10^9^/L. Spleen ultrasound was repeated without any abnormalities. He was discharged from the hospital on the seventh day following his treatment, having achieved full recovery. There were no complications, such as intra-abdominal hemorrhage, pancreatic fistula, or perforation of gastrointestinal organs, were observed during hospitalization. The resected cyst tissue was viewed as irregular pieces of tissue with grayish-red and smooth surfaces, and part of the area was cystic wall-like, with a smooth inner wall and a wall thickness of approximately 0.1–0.2 cm, The rest of the tissue was dark red on the cut surface (Figure 3A). Pathohistological examination revealed primary (epithelioid) cysts lined with a single layer of cuboidal or flat epithelium (Figure 3B), fibrous tissue hyperplasia with focal calcification, and no heterogeneous or malignant tumors (Figure 3C). The immunohistochemical results were positive for cytokeratin (CK) (Figure 3D). At the 3-month postoperative follow-up, no recurrence of the splenic cyst was seen on repeat abdominal ultrasound; blood cell analysis revealed an erythrocyte count of 10.14 × 10^12^/L and a platelet count of 298 × 10^9^/L (Figure 4).

## 3 Discussion

Primary epithelioid splenic cysts, also known as congenital splenic cysts, are usually asymptomatic in patients, and most of them are detected incidentally by imaging studies (12). In our case, the child was asymptomatic and was incidentally detected via abdominal ultrasound. In addition, the relatively rare clinical manifestations of splenic cysts may be associated with hematologic disorders such as thrombocytopenia, granulocytopenia, anemia, and other disorders (13). The detailed pathogenesis of primary splenic cysts is unclear, and three theoretical hypotheses exist (14, 15): (1) The spleen was invaded by the mesothelial endothelium during development, which is pluripotent in nature and can undergo chemotaxis to secrete fluids that cause the formation of cysts. (2) The lymphatic interstitial space theory, in which cysts may originate from the normal lymphatic interstitial space of the spleen. (3) Endodermal inclusion body theory, which proposes that epithelial splenic cysts are generated by the development of the chemotaxis of ectopic endodermal inclusion bodies within the spleen. However, some studies have reported that the nature of epithelioid cells may be due to the differentiation of teratomas or the presence of contain fetal squamous epithelial cell linings rather than from chemotaxis.

The epithelial cells of primary splenic cysts may secrete CA19-9 or CEA; therefore, elevated levels of the serum tumor markers CA19-9 and CEA are informative for the classification of splenic cysts (16); however, elevated levels of CA19-9 and CEA are most commonly observed in gastrointestinal tumors and are of limited value in identifying other types of splenic cystic lesions (17). In our case, no abnormalities in serum tumor markers were observed. Unfortunately, owing to limited conditions, we can't completely exclude the possibility of parasitic splenic cysts before surgery. Splenic cysts with a diameter greater than 14 cm were called giant splenic cysts. In our patient, the splenic cyst was 11 cm in diameter, which was close to the criteria for giant splenic cysts (18). Ultrasonography, CT or MRI are the most commonly used imaging methods in the diagnosis of splenic cysts (19). Ultrasonography usually reveals hypoechoic or anechoic lesions with well-defined borders within the parenchyma, cysts containing debris may exhibit varying degrees of internal echogenicity. CT imaging can further delineate the boundaries of the splenic cyst, which may exhibit internal septations. In our case, the child was admitted to the hospital with a high suspicion of a splenic cyst on both the abdominal ultrasound and the abdominal CT. On the basis of the patient's medical history, physical examination results and ancillary investigations, we considered the possibility that the of primary splenic cyst in the child was high. However, it is often necessary to identify the various diseases that can cause splenomegaly (4). Mild splenomegaly can be caused by infectious diseases such as mononucleosis, tuberculosis, congenital syphilis, histoplasmosis and septicaemia. Moderate splenomegaly is common in hematologic disorders, such as congenital hemolytic anemia and lymphoma. Severe splenomegaly is observed in leukemia, primary tumors (hemangiomas and lymphangiomas), and malaria. It also needs to be differentiated from duplicate malformations of the digestive tract (20). In fetuses or infants, most congenital splenic cysts are benign and do not cause harm. Fetal splenic cysts are mainly differentiated from cystic masses in the left upper abdomen, which can originate from the urinary system, reproductive system, adrenal glands, or gastrointestinal tract disorders (21).

Some scholars analysed the data of 16 patients with splenic cysts and reported that asymptomatic small cysts (less than 5 cm) have the possibility of disappearance and do not require any treatment, and require regular follow-up (15). Gezer et al. (22) retrospectively analysed 22 patients with splenic cysts in children, and reported that the cysts of 12 patients with asymptomatic and/or small cysts (diameter of 0.5–4.0 cm) completely disappeared after ultrasonographic examination and physical examination for 5 years of follow-up, 4 patients had complete disappearance of the cysts, 6 patients had no change in the cysts, and only 2 cysts appeared to be enlarged. Therefore, conservative treatment is recommended for asymptomatic and small splenic cysts. If a splenic cyst becomes symptomatic or has a diameter of more than 5 cm with a risk of infection, rupture, or hemorrhage, surgical treatment should be performed (23). Currently, total splenectomy is still considered the standard of care for the treatment of splenic cysts, which prevents recurrence and abdominal bleeding or infection; however, there is a risk of post total splenectomy sepsis (24). Costi et al. (25), in a study of splenectomy for the treatment of splenic cysts, reported that sepsis could occur in 0.2%–0.5% of total splenectomies, with a mortality rate of up to 60%. Maeda et al. (26) noted that the incidence of sepsis after total splenectomy ranged from 0.23% to 4.2%, with a mortality rate ranging from 38% to 69%. Therefore, to protect the immune function of the spleen, spleen-preserving surgery has become the main method of surgical treatment for splenic cysts. Partial splenectomy with preservation of more than 25% of the splenic parenchyma can achieve the goal of eradication, but also preserves the function of the spleen and avoids post total splenectomy septicemia, which is a better surgical choice (19). In our case, we actively refine preoperative evaluations and assessments to exclude contraindications for surgery in pediatric patients. Considering the patient's young age, which was only 13 years old, in our case, the splenic cyst was located in the upper pole of the spleen, and the child underwent a partial splenectomy after our team discussion.

Since Delaitre first reported laparoscopic splenectomy in 1991, laparoscopic splenectomy has been accepted by more surgeons and has become the mainstay of surgical treatment for most splenic cysts (2). With the continuous development of minimally invasive laparoscopic surgical techniques, to protect splenic function is crucial in significantly reducing the incidence and mortality rates of opportunistic infections following splenectomy (27). Laparoscopic spleen-preserving surgery can be technically challenging. The objective of laparoscopic partial splenectomy is to preserve splenic function, but this approach carries surgical risks including bleeding, inadvertent splenectomy, and recurrence of the condition (28). Hassoun et al. (29) reported a recurrence rate of less than 2% for laparoscopic partial splenectomy for the treatment of splenic cysts, whereas laparoscopic splenic cyst removal resulted in a recurrence rate of approximately 50%. In their case report on giant splenic cysts, Lowrie et al. (27) reported that laparoscopic approach removal of primary giant splenic cysts is technically feasible and may have a lower recurrence rate than the conventional open surgical approach. In addition, the incidence of incisional hernia after laparoscopy is less than 1%, whereas the incidence of incisional hernia after open surgery is 11% (30). Laparoscopic partial splenectomy is also feasible for benign splenic cysts according to a study conducted by Chen et al. (31), in which 16 patients were preoperatively diagnosed with different types of splenic cysts. None of the patients were converted to open surgery and none of the patients required blood transfusions or experienced postoperative complications. Wang et al. (32) performed laparoscopic partial splenectomy in 11 patients with focal benign splenic cysts with preservation of splenic function. Therefore, laparoscopic surgery has the advantages of a shorter hospitalization time, less pain, faster recovery, fewer complications, and greater patient satisfaction, making it a safe and effective treatment modality (9). In view of the many advantages of laparoscopy described above, combined with the child's own situation, we chose laparoscopic partial splenectomy for the child. Of course, total splenectomy is still recommended for splenic cysts with less than 25% of the normal spleen in the remaining splenic parenchyma assessed preoperatively; splenic cysts located in the splenic hilum or splenic parenchyma close to the splenic macrovessels; multiple splenic cysts; and splenic cysts with complications such as splenic infection, rupture, hemorrhage, or infarction (33).

With the maturity of laparoscopic technology, the continuous updating of instruments, and the increasing demand of patients for minimally invasive and cosmetic surgery, single-site laparoscopic surgery has become a hotspot in the field of pediatric minimally invasive surgery for exploration and clinical application (34). How to minimize scarring is a common desire of surgeons and patients, especially children. The “natural channel” of the navel has gradually attracted attention (35). Transumbilical single-site laparoscopic splenectomy not only utilizes the natural folds of the umbilicus to make the surgical incision very hidden, with good cosmetic results, but also, reflects the concept of “no scar”. Moreover, the transumbilical approach to splenectomy also avoids abdominal infections caused by natural cavities such as the vagina or digestive tract. In our report, the use of transumbilical single-site laparoscopic-assisted partial splenectomy is safe and feasible, which can significantly reduce the incidence of complications such as incisional hernia and incisional infection and, at the same time, reduce the chance of postoperative abdominal organ adhesions to a large extent. This approach has the advantages of less trauma, fewer complications, quicker recovery, less pain, and shorter hospitalization time after surgery, and it is truly scarless at the umbilicus, with good aesthetic and cosmetic results.

In our case, the following experiences and techniques were used: (1) During spleen removal, the patient's left flank was slightly elevated to utilize gravity, facilitating smooth placement of the spleen into the specimen bag. (2) To minimize collisions and friction among the three cannulas inserted through the umbilicus, we increased the distance between them by extensively freeing and enlarging the subcutaneous tissue around the incision. Additionally, the observation and operative trocar placements were adjusted to one shallow and two deep holes to enhance intraoperative maneuverability. (3) A trocar with smaller ends was selected for instrument insertion. When needed, a “scissor-type” cross-operation with left- and right-handed instruments was utilized to reduce interference between external instruments. (4) A gastric tube was placed preoperatively to allow intraoperative gastric residue cleaning and to enlarge the surgical space by evacuating pneumoperitoneum. Moreover, suspending the side of the greater curvature of the stomach from outside the abdominal cavity with a silk thread provided optimal anatomical exposure. (5) For large splenic cyst, aspirating the cystic fluid with a needle before removal can be advantageous. Care must be taken to prevent fluid leakage during this process. (6) During surgery, secondary splenic vessels should be carefully separated while avoiding excessive dissection of the perisplenic ligament. It is crucial to monitor the blood supply to the remaining spleen to prevent ischemic infarction postoperatively. Particular attention must be given to the short gastric vessels when dealing with the upper pole mass of the spleen; dissection of these vessels is necessary to prevent increased bleeding and other complications. In our case, the preoperative CT revealed no significant blood vessels in the splenic cyst, allowing us to use Ligasure for direct resection. (7) Removing the spleen through a 2 cm incision proved challenging. We utilized the spleen's gravity, and used grasping forceps to load the spleen into the specimen bag. The specimen bag was then lifted to the umbilical incision. Care was taken not to exert excessive force when lifting the specimen bag to avoid rupture, which could contaminate the abdominal cavity.

## 4 Conclusions

In conclusion, splenic cysts in children are uncommon, and the decision for surgical intervention depends on cyst size and the presence of symptoms. Asymptomatic cysts with a diameter less than 5 cm can be monitored with regular imaging. In contrast, cysts 5 cm or larger, or those causing symptoms, necessitate surgical treatment. To minimize serious complications associated with total splenectomy and satisfy aesthetic concerns for both children and parents, transumbilical single-site laparoscopic partial splenectomy offers a minimally invasive, effective, and cosmetically favorable option.

## Data Availability

The original contributions presented in the study are included in the article/Supplementary Material, further inquiries can be directed to the corresponding author.
